# Notes and News

**Published:** 1903-09-12

**Authors:** 


### NOTES AND NEWS.

The Duchess of Albany has consented to lay the memorial stone of the Royal Waterloo Hospital for Children and Women on October 22nd.

The first French Anti-alcohol Congress will be held in Paris in October. Mr. Casimir-Périer will preside.

The Adelaide City Council have put into force a by-law inflicting fines for expectoration on the pavements of the city.

The medical profession in Germany is terribly overcrowded, and the majority of its members have a struggle for existence. Yet the number of students ready to join the ranks continues unabated.

The holiday homes at Addiscombe, Croydon, erected by the late Lady Ashburton, have been handed over to the Ragged School Union. The accommodation is for sixty inmates, and the institution will be utilised as a holiday home for crippled children.

Amongst the English delegates attending the International Congress of Hygiene at Brussels were Professor Sims Woodhead, Sir James Creighton Browne, Sir Patrick Manson, and Dr. Dawson Williams. A lengthy and animated discussion on the subject of the transmission of bovine tuberculosis took place, and if no unanimous conclusion was arrived at, the meeting at any rate were in accord in agreeing that no precautions should be relaxed to safeguard human beings against any possible contagion. A resolution put by Sir Patrick Manson urging all Governments to take steps to recognise the mosquito malarial theory, and to enforce its adoption, was carried. Dr. Martin, of the Pasteur Institute in Paris, read an interesting paper on infantile diphtheria and the serum cures. The outcome of a discussion on the plague resulted in the passing of a resolution that the conditions of the quarantine regulations should be improved.

Nearly 100 tons of fish were seized last week at Billingsgate as unfit for food.

The Surgical Aid Society have presented artificial limbs to the man named Barry who was recently run over on the railway at Hull, which necessitated the amputation of his legs and arms.

Cholera has severely devastated the 2nd Battalion of the King's Shropshire Light Infantry at Ranikhet, Bengal. Twenty-five members have been attacked. The deaths amount to seventeen, including one officer.

The foundation stone of a new asylum for 570 patients has been laid near Bromsgrove for the county of Worcester. The total cost is estimated at £216,000. The present County Aslyum for 1,000 inmates does not provide sufficient accommodation.

A Scottish Sanitary Congress was held last week at Stranraer, when some interesting papers were read and useful discussions took place on several topics of general utility, such as the milk supply of large towns, the provision of isolation hospitals, infant mortality, and other subjects.

For failing to notify a case of small-pox at Burton-on-Trent, a local medical man has been fined 40s. and costs. At least 19 cases have been traced as directly resulting from infection from this one patient. Medical men labour under considerable difficulty in diagnosing small-pox, for a very large number have never acquainted themselves personally with the aspects of the disease. A few more convictions through mistaken diagnosis may render all alive to the necessity of completing their knowledge in this direction.

It is reported that the plague has broken out at Marseilles, and that eleven deaths have taken place, all occurring amongst Italian workmen occupied in a manufactory to which the infection is believed to have been conveyed by means of a consignment of rags from Constantinople. A hospital has been set apart for the reception of the plague cases.

It is interesting to compare the methods of precaution against infection as carried out in Chicago and in Lincoln. The severity of the means resorted to in Chicago are set forth in our annotation on the subject, and are shown to have proved but partially successful. The authorities in Lincoln, apparently quite as much in earnest as those of Chicago, adopted the reward rather than punishment system of prevention. The contacts in a lodging-house infected with small-pox were invited to take up their abode in a comfortable tent, where good food and service was provided, and a sum of money offered to each to compensate for a fortnight's detention and isolation. The contacts appear to have willingly fallen in with the arrangements. Such a system, though it may appear extravagant, if effectual in preventing one case from starting an epidemic, is really very economical. Chicago can show that the strictest law can defeat its object, and Lincoln shows that persuasion has a better chance.

